# Long Noncoding RNA Identification: Comparing Machine Learning Based Tools for Long Noncoding Transcripts Discrimination

**DOI:** 10.1155/2016/8496165

**Published:** 2016-11-29

**Authors:** Siyu Han, Yanchun Liang, Ying Li, Wei Du

**Affiliations:** ^1^College of Computer Science and Technology, Key Laboratory of Symbol Computation and Knowledge Engineering of Ministry of Education, Jilin University, Changchun 130012, China; ^2^Zhuhai Laboratory of Key Laboratory of Symbol Computation and Knowledge Engineering of Ministry of Education, Zhuhai College of Jilin University, Zhuhai 519041, China

## Abstract

Long noncoding RNA (lncRNA) is a kind of noncoding RNA with length more than 200 nucleotides, which aroused interest of people in recent years. Lots of studies have confirmed that human genome contains many thousands of lncRNAs which exert great influence over some critical regulators of cellular process. With the advent of high-throughput sequencing technologies, a great quantity of sequences is waiting for exploitation. Thus, many programs are developed to distinguish differences between coding and long noncoding transcripts. Different programs are generally designed to be utilised under different circumstances and it is sensible and practical to select an appropriate method according to a certain situation. In this review, several popular methods and their advantages, disadvantages, and application scopes are summarised to assist people in employing a suitable method and obtaining a more reliable result.

## 1. Introduction

Long noncoding RNAs (lncRNAs), one of the most poorly understood but also the most common RNA species, are those noncoding transcripts with length more than 200 nucleotides. Initially, people classified noncoding RNA (ncRNA) genes as “junk gene” or transcriptional “noise” [1]. Nonetheless, researchers found that about 70% of the genome is transcribed in various contexts and cell types [2, 3], about 80% of the genome has biochemical functions [4], and many DNAs code for RNAs as the end products instead of proteins [5]. LncRNAs are involved in a wide range of cellular mechanisms such as the regulation of genome activity [6], histone modifications [7, 8], and DNA methylation [9]. In addition, lots of studies have demonstrated that lncRNAs have a significant role in diverse biological processes; thus lncRNAs are especially important to the studies of human biology and diseases [10]. For example, in prostate cancer of human, lncRNA* SChLAP1* and chromatin remodelling complex SWI/SNF have opposing roles.* SchLAP1* has an interaction with the SNF5 subunit of SWI/SNF and inhibits binding of SWI/SNF to chromatin, which leads to genome-wide derepression of gene activity [11]. Moreover, aberrant expression of lncRNAs in cancer can be regarded as biomarkers and therapeutic targets because of its extremely specific expression [6]. The LncRNADisease database now integrates more than 1000 lncRNA-disease entries and 475 lncRNA interaction entries, which suggested that lncRNAs are associated with diseases closely [12].

Since lncRNAs so closely interact with diseases, many lncRNA-disease association detection tools are invented. Assuming that lncRNAs with similar functions tend to associate with similar diseases, a semisupervised method, Laplacian Regularized Least Squares for LncRNA-Disease Association (LRLSLDA) [13], was developed; this tool displays a satisfying result and needs no negative samples. Nonetheless, this method is facing the problems of parameter selection and classifier combination. The principal idea of LRLSLDA, as mentioned above, is to measure the functional similarity of lncRNAs, which means that the performance of similarity calculation model largely determines the performance of association model. The similarity calculation model of LRLSLDA is LFSCM (LncRNA Functional Similarity Calculation based on the information of MiRNA) which is based on lncRNA-miRNA interactions and miRNA-disease associations. In 2015, novel lncRNA functional similarity calculation models (LNCSIM) [14] were provided by Chen et al. By integrating LRLSLDA and LNCSIM, the performance was enhanced. Recently, a new lncRNA functional similarity calculation model, FMLNCSIM (Fuzzy Measure-Based LncRNA Functional Similarity Calculation Model) [15], has been developed; this new model has a web interface (http://219.219.60.245/) for users' convenience. Considering that nowadays the experimentally confirmed data of miRNA-disease associations are much easier to obtain than the ones of lncRNA-disease, Chen [16] utilised the miRNA-disease association and miRNA-lncRNA interaction to identified lncRNA-disease association. This method (HGLDA) circumvents the utility of LncRNADisease database but still presents the desired results. Currently, many other tools, such as RWRlncD [17] and RWRHLD [18], were designed aiming at predicting lncRNA-disease association and obtaining more reliable results. Unfortunately, they have their own limitations [16]. As the titles of these methods implied, RWRlncD and RWRHLD mainly predict the association by utilising Random Walk with Restart (RWR). RWRlncD can only be applied to the case that lncRNAs have known related diseases and RWRHLD cannot deal with the circumstance that lncRNAs have unknown lncRNA-miRNA interactions. Another method, Improved Random Walk with Restart for LncRNA-Disease Association (IRWRLDA) [19], is also based on RWR, but IRWRLDA can predict the associations even when diseases show no known related lncRNAs.

Research [20] has illustrated the lncRNA-disease association extensively and comprehensively. Basically, there are three approaches to performing lncRNA-disease association prediction [20]: to train a model based on machine learning algorithm; to construct a heterogeneous network; or to integrate lncRNA-miRNA interactions and miRNA-disease associations. Currently, researches have acknowledged that it is imperative to analyse the role of lncRNAs in many diseases especially cancer, but the first step and fundamental work is how to discriminate lncRNAs from genes. With the rapid development of next-generation sequencing technologies, thousands and thousands of transcriptomes have been discovered, which furnished us with more and more useful information on ncRNAs. Meanwhile, many ncRNAs identification approaches have been developed to facilitate the researches and analyses. Each kind of ncRNA has its own prediction tools such as tRNAscan-SE (1997) [21] and tRNA-Predict (2015) [22] for transfer RNA (tRNA) identification; mirnaDetect (2014) [23] and imDC (2015) [24] for microRNA (miRNA) prediction; and RNAmmer (2007) [25] for ribosomal RNA (rRNA) discrimination. Both tRNA-Predict and mirnaDetect are constructed with the features of secondary structure and codon-bias. The method imDC is an algorithm of ensemble learning to deal with imbalanced data and is applied to miRNA classification. The research area of ncRNA is fast growing. However, it is still a challenge to distinguish lncRNAs from protein-coding genes in that lncRNAs share many features similar to mRNAs. Moreover, the incomplete transcripts or genes poorly annotated or containing sequencing errors also thwart the discrimination and functional inference. During the last ten years, many efforts on lncRNA identification have been made and many approaches have been developed to make a more accurate discrimination. Several studies [26, 27] have summarised and reviewed the approaches of ncRNAs identification and analysis, but a few report the discussion of lncRNAs prediction methods. Wang et al. [26] discussed several ncRNA detection methods based on homology information and common features. Different approaches aiming at detecting different kinds of ncRNAs are presented and an overview of some useful tools was given, yet no analysis on application scopes was provided. Hence, the summary of these methods is more theoretical than practical. Veneziano et al. [27] summarised some computational approaches of ncRNA analysis based on deep sequencing technology. Some lncRNA prediction tools were discussed briefly but many other helpful tools were excluded.

In this paper, we mainly focus on the tools for lncRNA identification. The aim of this paper is to summarise the popular algorithms of lncRNA identification and to assist researchers in determining which method is more appropriate for their purpose. Here, comprehensive analyses and discussions of these tools were provided. Then, we compared several popular machine learning based methods, including Coding Potential Calculator (CPC) [28], Coding Potential Assessment Tool (CPAT) [29], Coding-Non-Coding Index (CNCI) [30], predictor of long noncoding RNAs and messenger RNAs based on an improved* k*-mer scheme (PLEK) [31], Long noncoding RNA IDentification (LncRNA-ID) [32], and lncRScan-SVM [33]. In addition, lncRNA-MFDL [34] and LncRNApred [35], two artificial neural network- (ANN-) involved tools, are also introduced in this paper. However, the provided access link of lncRNA-MFDL has been forbidden; LncRNApred often throws errors while handling massive-scale data which can be processed by CPC and CPAT successfully. Thus, we only briefly introduce the algorithms of the classification model but omit the discussions of application scope. We expect that this review can be a practical manual when readers conduct lncRNA identification researches.

CPC (2007) is used to assess the protein-coding potential of transcripts with high accuracy and speed [28]. However, with the emergence of new programs, speed is scarcely considered as a merit. The features of CPC can be divided into two categories. The first one is based on the extent and quality of the Open Reading Frame (ORF), and the other category is derived from BLASTX research. The authors employed the LIBSVM package to train support vector machine (SVM) model with the standard radial basis function kernel [36].

CPAT (2013) is another protein-coding potential assessment tool based on the model of logistic regression. The selected features include the quality of the ORF, Fickett Score, and hexamer score. Fickett Score is used to evaluate each base's unequal content frequency and asymmetrical distribution in the positions of codons in one sequence. Hexamer score is mainly based on the usage bias of adjacent amino acids in proteins.

CNCI (2013) is a classifier to differentiate protein-coding and noncoding transcripts by profiling the intrinsic composition of the sequence. According to the unequal distribution of adjoining nucleotide triplets (ANT) in two kinds of sequences, a 64*∗*64 ANT Score Matrix is constructed to evaluate the sequence and the sliding window is used as a supplement to achieve a more robust result [30]. ANT bears some similarities to the hexamer score of CPAT, but much more comprehensive and intricate analysis was conducted to facilitate the incomplete transcripts classification. The classification model of CPAT is SVM with a standard radial basis function kernel.

PLEK (2014) uses* k*-mer scheme and sliding window to analyse the transcripts. For multiple species, PLEK does not have too many advantages over CNCI on testing data of normal sequence. Nevertheless, compared with PLEK, the results of CNCI will deteriorate when the sequence contains some insert or deletion (indel) errors. These errors are very common in today's sequencing platforms. The classification model of PLEK is SVM with a radial basis function kernel.

LncRNA-ID (2015) has 11 features which can be categorized according to ORF, ribosome interaction, and the conservation of protein. The first category is similar to the ORF features in CPC and CPAT. The foundation of the second feature category is the interactions between mRNAs and ribosomes during protein translation since some studies displayed that lncRNAs can be associated with ribosomes [37, 38] but do not show the release of ribosomes [39]. The profile hidden Markov model-based alignment is used to assess the conservation of protein. The classification model of LncRNA-ID is improved using random forest which assists LncRNA-ID effectively in handling imbalanced training data.

Some tools are initially designed to predict ncRNAs but can also be applied to lncRNAs prediction, such as Phylogenetic Codon Substitution Frequencies (PhyloCSF, 2011) [40] and RNAcon (2014) [41]. Based on nucleotide substitutions and formal statistical comparison of phylogenetic codon models [40], PhyloCSF utilises multiple sequence alignments to find conserved protein-coding regions. As an alignment-based method, PhyloCSF entails high-quality alignments and suffers from low efficiency. RNAcon mainly predicts ncRNAs utilising* k*-mer scheme. Based on graph properties [41, 42], RNAcon can also perform ncRNAs classification and classify different ncRNA classes.

Some methods are especially developed for long intergenic noncoding RNAs (lincRNAs, one subgroup of lncRNAs) classification, such as iSeeRNA (2012, web server and Linux binary package available at http://137.189.133.71/iSeeRNA/index.html) [43] and LincRNA Classifier based on selected features (linc-SF, 2013) [44]. iSeeRNA built a SVM model with three feature groups: ORF; adjoining nucleotides frequencies (GC, CT, TAG, TGT, ACG, and TCG); and conservation score obtained from Phast [45]. The classifier of linc-SF evaluates the sequences with the criteria of sequence length, GC content, minimum free energy (MFE), and* k*-mer scheme.

## 2. Details of the Methods

In this part, we will discuss the machine learning models and the selected features of each method more specifically. Firstly, for users' convenience, some brief information of each method is displayed in Table 1 and the details of using are summarised. Then the details of each method are provided in the following.  Table 2 is a summary about the features selected by each method.

### 2.1. Details of Using

CPC can be downloaded from http://cpc.cbi.pku.edu.cn/download/. CPC has a user-friendly web interface at http://cpc.cbi.pku.edu.cn/programs/run_cpc.jsp. Documents and User Guide are provided at the website. To run CPC on a local PC, a comprehensive protein reference database is required and users can download it from ftp://ftp.ncbi.nlm.nih.gov/blast/db/ or ftp://ftp.uniprot.org/pub/databases/uniprot/uniref/uniref90/. About 20 gigabytes (GB) of free space is also needed for storing the protein reference database.

CPAT is also available both for download and as a webserver. Users can obtain the latest resource code from https://sourceforge.net/projects/rna-cpat/files/?source=navbar. Prereleases, tutorial files, and examples are also supplied on the pages. CPAT requires Python 2.7.x; numpy; cython; and R when running offline. The web server is available at http://lilab.research.bcm.edu/cpat/index.php.

CNCI can be downloaded at https://github.com/www-bioinfo-org/CNCI. Version 2 is updated on Feb 28, 2014. Setup and running steps are attached on the websites. Libsvm-3.0 has been enclosed in the package. Other additional files can be downloaded at http://www.bioinfo.org/np/.

PLEK was implemented by C and Python. The source code can be freely downloaded from https://sourceforge.net/projects/plek/files/. Several videos to assist user in utilising PLEK correctly are also provided. Python 2.7.x is required.

Scripts of LncRNA-ID can be obtained at https://github.com/zhangy72/LncRNA-ID.

LncRScan-SVM provided scripts, gene annotation files, and datasets. The scripts can be downloaded at https://sourceforge.net/projects/lncrscansvm/?source%20=%20directory. A Readme file is also attached on this site.

All the stand-alone versions of these tools require Linux/UNIX operating system.

The link of lncRNA-MFDL provided is https://compgenomics.utsa.edu/lncRNA_MDFL/. LncRNApred only has the web interface and is available at http://mm20132014.wicp.net:57203/LncRNApred/home.jsp. However, the link of lncRNA-MFDL expired when we did this research. And LncRNApred only provides a web server which cannot handle too many sequences at one time.

### 2.2. CPC in Detail

CPC [28] extracted six features to evaluate the coding potential of transcripts. Log-odds score, coverage, and integrity of ORF are used to assess the ORFs of one sequence. ORFs are predicted by* framefinder*. A high-quality ORF tends to have a high log-odds score and a larger ORF coverage. The integrity of ORF means ORFs in protein-coding transcripts are disposed of to begin with a start codon and end with a stop codon. The other three features are number of hits, hit score, and frame score, which are derived from the output of BLASTX search. A protein-coding transcript prefers more hits in alignment with lower *E*-values. Then the hit score is defined as follows [28]:

(1) Si=meanj−log10 Eij,i∈0,1,2,Hit  Score=meani∈0,1,2Si=∑i=02Si3,

where *E*_*ij*_ is the *E*-Value of the *j*th hits in the *i*th ORF. A noncoding transcript may also contain some hits, but these hits are inclined to scatter in three frames rather than be located in one. The frame score to calculate the distribution of hits among three ORFs is defined in the following:

(2) $$\begin{array}{c} Frame\;\;Score=\underset{i\in \left\{0,1,2\right\}}{variance}\left\{\;S_{i}\;\right\}=\frac{\sum_{i=0}^{2}{\left(S_{i}-\bar{S}\right)}^{2}}{2}. \end{array}$$

Thus, a protein-coding transcript will achieve a higher hit score and frame score because of the lower *E*-value and biased distribution of the hits.

The training data of CPC [46] are eukaryotic ncRNAs from the RNAdb [47] and NONCODE [48, 49] databases. CPC is designed to assess transcripts' protein-coding potential, which means it will have high accuracy of discriminating protein-coding transcripts. Moreover, CPC also has the error tolerance capacity, which owes much to* framefinder*'s accurate prediction.* Framefinder* performed well even though input transcripts may have some point mutations, indel errors, and truncations. CPC is slightly inferior in distinguishing noncoding transcripts in respect of the fact that lncRNAs may contain putative ORFs and transcript length is also familiar to protein-coding transcripts. The slow speed is another imperfection of CPC.

### 2.3. CPAT in Detail

CPAT [29] is an alignment-free program. CPAT uses a logistic regression model and can be trained on own data of users. Apart from the features of maximum length and coverage of ORF akin to CPC, Fickett Score is another criterion. Fickett Score can be regarded as a dependent classifier; it is mainly based on calculating the position of each base favoured and the content of each base in the sequence [50]. The base's position parameter of CPAT is defined as follows:

(3) A1=Number  of  As  in  positions  0,3,6,…A2=Number  of  As  in  positions  1,4,7,…A3=Number  of  As  in  positions  2,5,8,…A-position=max⁡A1,A2,A3min⁡A1,A2,A3+1,A-content=Occurence  Number  of  ATotal  Number  of  all  bases,

where *A* in the formula means the base *A* and the other three bases are measured in a similar way. The parameter of position calculates each base's favoured position and the parameter of content is the percentage of each base in the sequence. Then according to distributions of eight parameters' values [50], it is easy to obtain the probability that the sequence will be a protein-coding transcript. Next, each probability is multiplied by a weight to make a more accurate result. The weight is the percentage of the times that the estimate of each parameter alone is correct. Finally, according to the above descriptions, Fickett Score can be determined as follows:

(4) $$\begin{array}{c} Fickett\;\;Score=\overset{8}{\underset{i=1}{\sum }}p_{i}w_{i}. \end{array}$$

According to Fickett [50], Fickett Score alone can correctly discriminate about 94% of the coding segments and 97% of the noncoding segments with 18% of “No Opinion.”

The last feature of CPAT is hexamer score, which is the most discriminating feature. Hexamer means the adjacent amino acids in proteins. The features of the in-frame hexamer frequency of coding and noncoding transcripts are calculated and hexamer score is defined in the following:

(5) $$\begin{array}{c} Hexamer\;\;Score=\frac{1}{m}\overset{m}{\underset{i=1}{\sum }}\log\left(\;\frac{F\left(H_{i}\right)}{F^{\prime }\left(H_{i}\right)}\;\right). \end{array}$$

There are 64*∗*64 kinds of hexamers, and *i* denotes each hexamer. *F*(*H*_*i*_) (*i* = 0,1, 2,…, 4095) means the in-frame hexamer frequency of protein-coding transcripts, while *F*′(*H*_*i*_) means noncoding transcripts. For a transcript containing *m* hexamers, a positive hexamer score indicates a protein-coding transcript.

A high-quality training dataset is constructed containing 10,000 protein-coding transcripts selected from RefSeq database with the annotations of the Consensus Coding Sequence project and 10,000 noncoding transcripts randomly collected from GENCODE database. CPAT is prebuilt hexamer tables and logit models for human, mouse, fly, and zebrafish. Meanwhile, CPAT uses pure linguistic features to facilitate discrimination of the poorly annotated transcripts. CPAT has an efficient offline program and also provides a user-friendly web interface.

### 2.4. CNCI in Detail

CNCI [30] is mainly based on sequence intrinsic composition, it evaluates the transcripts by calculating the usage frequency of adjoining nucleotide triplets (ANT). Firstly, two ANT matrices are constructed based on the usage frequency of ANT in noncoding sequences and coding region of the sequences (CDS). For 4,096 ANT, the formulas to calculate each ANT usage frequency are defined as follows:

(6) XiN=∑j=1nSjXi,T=∑i=1mXiN=∑i=1m ∑j=1nSjXi;m=64×64;  n=1,…,N,XiF=XiNT,

where *X* means one kind of ANT; *S*_*j*_(*X*_*i*_) is the occurrence number of *X*_*i*_ in one sequence *S*_*j*_. Thus, *X*_*i*_ *N* denotes the total occurrence number of one kind of ANT in the dataset while *T* indicates the total occurrence number of all kinds of ANT in the dataset. Accordingly, *X*_*i*_ *F* is the usage frequency of ANT. Then the ANT Score Matrix is utilised, which is the log_2_-ratio of the two above-mentioned ANT matrices, to score a sequence and make a discrimination.

(7) $$\begin{array}{c} \text{ANT Score Matrix}={log}_{2}\frac{\text{CDS Matrix}}{\text{Non-coding Matrix}}. \end{array}$$

The distinguishing results of ANT Score Matrix are fairly well, but the matrix is constructed by computing the ANT usage frequency of coding region and noncoding region; consequently the untranslated region (UTR) of the entire sequence will interfere with the performance of discrimination. The sliding window is employed with one ANT (3 nt) in each scan step to identify the CDS of a sequence by scanning six reading frames of each sequence. The different sizes (30,60,90,…, 300 nt) of the sliding windows are examined and the size of 150 nt for this classification model is found to obtain the most robust result. For a sequence consisting of *k* ANT, there will be *k* − 1 segments in this sequence. Based on the ANT Score Matrix, each segment will get an *S*-Score, and each reading frame can obtain an array comprised of the *S*-Scores. The formula of *S*-Score is defined as follows:

(8) $$\begin{array}{c} S\text{-Score}=\overset{n}{\underset{i=1}{\sum }}\left\{\;H_{p}\left(\;X_{i}\;\right)\;\right\}, \end{array}$$

where *X* means ANT, *H*_*p*_ is the ANT Score Matrix, and *n* is the total number of the ANT in one segment or the whole sequence. Hence, a correct reading frame of coding transcript tends to have a higher whole sequence *S*-Score and, in this array of reading frame, the region composed of consecutive high *S*-Scores is the CDS. For long noncoding transcripts, the Maximum Interval Sum [51] program is used to identify the most-like CDS (MLCDS) which is the region that gained the largest sum of consecutive *S*-Scores in each reading frame. Among those six MLCDS, the length and *S*-Score of the MLCDS with the highest value are selected as the features of CNCI. Furthermore, the features of the LENGTH-Percentage, SCORE-Distance, and codon-bias are also selected to improve accuracy:

(9) LENGTH-Percentage=M1∑i=0nYi,SCORE-Distance=∑j=0nS−Ej5,

where *M*1 is the length of the MLCDS with the highest *S*-Score, *Y*_*i*_ is the length of each MLCDS, *S* is the highest *S*-Score among six MLCDS, and *E*_*j*_ is the *S*-Score of other five MLCDS. Codon-bias (3-mer frequencies) is a parameter to evaluate the usage bias of different codons in protein-coding or long noncoding transcripts. The log_2_-ratio of occurrence frequency of each codon (stop codons are excluded) in protein-coding genes and lncRNAs is calculated, and most codons have distinct usage bias in two kinds of sequences.

The training datasets of CNCI contain protein-coding transcripts selected from RefSeq database and long noncoding transcripts selected from GENCODE [52]. The CNCI is applied to other species with the aim of examining the scope of application. The results of vertebrates (except birds), especially mammals, can be accepted since the program was trained on human gene set. CNCI can be used to discriminate incomplete transcripts, especially those high-throughput sequencing data of poorly explored species.

### 2.5. PLEK in Detail

PLEK [31] is an alignment-free tool based on *k*-mer frequencies of the sequences. For a given sequence, the sliding windows with size of *k* scan 1 nt as a step forward. *k* ranges from 1 to 5, which is a trade-off between accuracy and computational time. Thus, for a sequence consisting of *A*, *C*, *G*, and *T*, the 4^1^ + 4^2^ + 4^3^ + 4^4^ + 4^5^ = 1,364 patterns can be obtained. Then the following formulas can be used:

(10) fi=ciskwk,k=1,2,3,4,5;  i=1,2,…,1364,sk=l−k+1,wk=145−k,k=1,2,3,4,5,

where *i* is the number of the patterns; *c*_*i*_ denotes the number of the segments in sliding windows matching with patterns; *s*_*k*_ denotes the total of the segments when sliding window slides along the sequence with the size of *k*. Therefore, *f*_*i*_ is the usage frequency multiplied by a factor *w*_*k*_ which is used to facilitate the discrimination.

A balanced training dataset is conducted with all 22,389 long noncoding transcripts collected from the GENCODE dataset [52–54] and 22,389 protein-coding transcripts randomly selected from the human RefSeq dataset [55, 56]. Though the training model of PLEK is human, PLEK can still be applied to other vertebrates. PLEK is particularly designed for the transcripts acquired from current sequencing platforms which consist of some indel errors commonly. For these transcripts, the performance of PLEK is better than CPC and CNCI. PLEK can be trained with users' own datasets, but it may take a long time to be accomplished.

### 2.6. LncRNA-MFDL in Detail

LncRNA-MFDL [34] is based on feature fusion and deep learning algorithm. LncRNA-MFDL has four kinds of features which are integrated to build a classification model based on deep stacking networks (DSNs, one kind of deep learning algorithm) [57, 58]. Four feature groups of lncRNA-MFDL include *k*-mer; secondary structure; ORF, obtained by utilising txCdsPredict program (http://genome.ucsc.edu/) [59]; and MLCDS features which are inspired by CNCI [30].

The *k*-mer scheme employed in lncRNA-MFDL is unlike the one in PLEK. Here, the *k* only ranges from 1 to 3, but the frequencies are calculated on the regions of the whole sequence and ORF at the same time. Considering that the secondary structure is more conserved and stable than primary structure, a representative criterion, the minimum free energy (MFE), is used to assess the secondary structure of the transcripts. Utilising RNAfold program of ViennaRNA Package [60], the MFE, the ratio of MFE to sequence length, and the number of paired bases and unpaired bases can be easily obtained.

### 2.7. LncRNA-ID in Detail

LncRNA-ID [32] has three categories of features as mentioned earlier. Except for the length and coverage of ORF, the features based on translation mechanism and protein conservation are extracted.

Many studies [61–63] have demonstrated that several nucleotide sites in Kozak motif play a prominent role during the initiation of protein translation. An efficient translation indicates that the highly conserved nucleotides appear at the positions {−3, +4} and {−2, −1} of Kozak motif GCC*R*CCAUGG (*R* represents purine and the position of A in start codon AUG is +1). Thus, these conserved sites are more likely to exist in protein-coding transcripts. Moreover when the translation starts, the binding energy will change along with the interaction between the 3′ end of rRNAs and mRNA transcripts. The Ribosome Coverage to calculate the changes of the binding energy is defined as follows:

(11) $$\begin{array}{c} \text{Ribosome Coverage}=\overset{L}{\underset{i=1}{\sum }}\left\{\;N_{i}\;|\;\delta_{i}<0\;\right\}, \end{array}$$

where *δ*_*i*_ is the free energy at position *i* and *N*_*i*_ is the number of base pairs starting at position *i* in a sequence with the length of *L*. Next, the three levels of ribosome occupancy by computing Ribosome Coverage on three regions, respectively, are obtained: the whole transcript, ORF, and 3′UTR. Accordingly, a true protein-coding transcript tends to attain higher Ribosome Coverage on the whole transcript and the ORF region. When the translation terminates, the ribosomes will be released from protein-coding transcripts. Therefore, it is likely to capture a considerable drop of ribosome occupancy when ribosomes reach stop codons. The Ribosome Release Score to capture this change of ribosome occupancy is defined:

(12) Ribosome  Release  Score=Ribosome  coverage  of  ORF/lengthORFRibosome  coverage  of  3′UTR/length3′UTR,

and a protein-coding transcript inclines to exhibit a higher Ribosome Release Score. For protein translation category, the selected features including nucleotides at two positions of Kozak motif, Ribosome Coverage on three regions, and Ribosome Release Score are selected.

The protein conservation of the sequences is evaluated according to profile hidden Markov model-based alignment scores. HMMER [64] is a software suite for sequence homology detection using probabilistic methods. LncRNA-ID employed HMMER with the *E*-value cutoff of 0.1 to align the transcripts against all available protein families. A protein-coding transcript is expected to get a higher score, longer aligned region, and a reasonable length of the profile in the alignment.

In human genome, although the amount of lncRNA is at least four times more than protein-coding genes [65], the majority class in training data is protein-coding transcript on account of poorly annotated lncRNA. Hence, the classification model of this method is balanced random forest [66, 67] which is derived from random forest but could utilise the sufficient protein-coding data and avoid inaccurate results caused by the imbalanced training data at the same time. The human prebuilt model of LncRNA-ID contains 15,308 protein-coding transcripts and 4586 lncRNAs from GENCODE [52]. For mouse, the training datasets are comprised of 22,033 protein-coding transcripts and 2,457 lncRNAs randomly selected from GENCODE. These two datasets were also used to draw receiver operation characteristic (ROC) curves in the next section (Figure 2). Users can train LncRNA-ID with their own dataset and apply it to various species.

### 2.8. LncRScan-SVM in Detail

LncRScan-SVM [33] classifies the sequences mainly by evaluating the qualities of nucleotide sequences, codon sequence, and transcripts structure. The counts and average length of exon in one sequence are calculated. The protein-coding transcripts are disposed of to include more exons, thus having a longer exon length than lncRNA. Another feature is the score of* txCdsPredict*. This third-part program from UCSC genome browser [68] can determine if a transcript is protein-coding. Conservation score is obtained by calculating the average of PhastCons scores [45] from Phast (http://compgen.cshl.edu/phast/). Transcript length and standard deviation of stop codon counts between three ORFs are the last two features.

The reliable datasets are constructed from GENCODE [54] composed of 81,814 protein-coding transcripts and 23,898 long noncoding transcripts of human. And, for mouse, 47,394 protein-coding transcripts and 6,053 long noncoding transcripts from GENCODE [52] are also contained within the dataset. After being trained on human and mouse datasets, lncRScan-SVM obtains a good performance on lncRNA prediction.

### 2.9. LncRNApred in Detail

Before constructing the classifier, self-organizing feature map (SOM) clustering [69] is employed to select representative samples as the training dataset, which enhanced the performance of LncRNApred. As to the features, the length and coverage of the longest ORF, one of the classical and typical features, are selected as the criteria. In addition, G + C content, *k*-mer (*k* is from 1 to 3 just like lncRNA-MFDL), and length of the sequence are also the features of LncRNApred. The novel idea of LncRNApred is SNR, which transforms one sequence into four binary numeric sequences:

(13) ub=1,Sn=b,0,Sn≠b,n=0,1,2,…,N−1,  b∈A,T,C,G,

where *b* means four kinds of bases, *N* is the length of one sequence, and *S*[*n*] denotes a sequence of length *N*. Thus, there will be four binary sequences {*u*_*b*_∣*b* ∈ (*A*, *T*, *C*, *G*)}. Then applying Discrete Fourier Transform (DFT) to these four binary numeric sequences, the power spectrum {*P*[*k*]} can be obtained:

(14) Ubk=∑n=0N−1ubne−i2πnk/N,k=0,1,…,N−1,Pk=UAk2+UTk2+UGk2+UCk2.

The studies of Fickett [50, 70] have presented that positions and compositions of four bases are different in lncRNAs and protein-coding RNA, and, because of this, the power spectrum of one protein-coding transcript will have a peak at *N*/3 position. Hence, the SNR is defined as follows:

(15) E−=∑k=0N−1PkN,SNR=PN/3E−.

Now, there are 89 features: the length and coverage of the longest ORF, the length of the sequence, SNR, G + C content, and 4 + 16 + 64 features of *k*-mer. Noticing that not all the features have high discriminative power, the feature selection is made and 25 high-quality features are determined from the original 84 features of* k*-mer. Finally, 30 features are selected to build a random forest model. The performance of random forest is largely determined by training set. Therefore, the clustering method is used to find out the most adequate sequences to form a high standard training set. The clustering method SOM [69] achieved the best result and was chosen to select characteristic sequences

**Figure 1:**
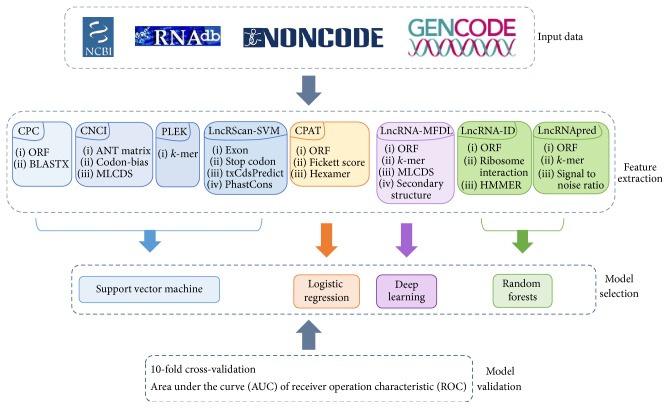
An overall procedure of eight tools. The features of each tool are sorted into several groups and only the categories of the features are listed in the figure.

An overall procedure of these eight tools is displayed in Figure 1.

## 3. Performance of These Methods

To quantify the classification performance under one unified standard, we first characterise lncRNAs as the positive class and protein-coding transcripts as the negative class; then the performance of these tools can be evaluated with several standard criteria defined as follows:

(16) Sensitivity=TPTP+FN,Specificity=TNTN+FP,Accuracy=TP+TNTP+FP+TN+FN,False  Positive  Rate=FPFP+TN.

As one of the most popular methods, CPC is especially designed for assessing protein-coding potential and performed fairly well for discriminating protein-coding transcript. It enjoys the best results when screening the coding transcripts. For 10,000 protein-coding genes and 10,000 lncRNAs selected from UCSC genome browser (GRCh37/hg19), CPC picked up about 97.62% coding transcripts while CPAT distinguished 85.28% of them. CPAT also outperforms CPC with 89.94% accuracy [33].  Table 3 shows the performance of these tools on the same testing dataset. CPC picks up 99.97% of human protein-coding genes collected from GENCODE, in comparison with the latest program LncRNA-ID whose performance is 95.28%. However, the performance of CPC appears to somewhat decline when focusing on the capability of discriminating noncoding transcripts, especially long noncoding transcripts: CPC only picked up 66.48% of human's long noncoding transcripts while the results of CPAT, PLEK, and LncRNA-ID are 86.95%, 99.52%, and 96.28%.

CPC and CPAT are the programs to assess the coding potential, but CNCI is especially used to classify protein-coding and long noncoding transcripts. With the sequences becoming longer and longer, CNCI was more superior to CPC. According to Sun et al. [30], when the length of transcript is longer than 2,000 nt, the accuracy of CPC is only around 0.4 while the CNCI still has an outstanding performance. The training dataset of CNCI is human but this method still achieved more than 90% accuracy in other vertebrates apart from the birds [30]. PLEK is tested on two datasets sequenced by PacBio and 454 platforms (refer to Table 3). Among the tools being compared, CPC still picked up about 99.90% coding genes though this figure is not that useful because it can only distinguish 19.00% and 47.20% lncRNAs. CNCI displayed better performance on both datasets, but PLEK even achieved a more satisfying result.

LncRNA-ID is another method to identify the long noncoding transcripts. Compared with other programs, LncRNA-ID strikes a good balance between sensitivity and false positive rate. According to Table 3, it is noticeable that lncRNA is better than PLEK but slightly inferior to CPC and CPAT on the testing data of coding genes, and the performance on lncRNAs is just the opposite. LncRNA-ID can be trained with users' own data; it can obtain a satisfying result even when the data is unbalanced. With the proportion of lncRNA decreasing, CPAT shows a sharp reduction from 79.51% to 54.46% on the capability of lncRNA discrimination; LncRNA-ID, by contrast, fell less than 1% [32].

**Figure 2:**
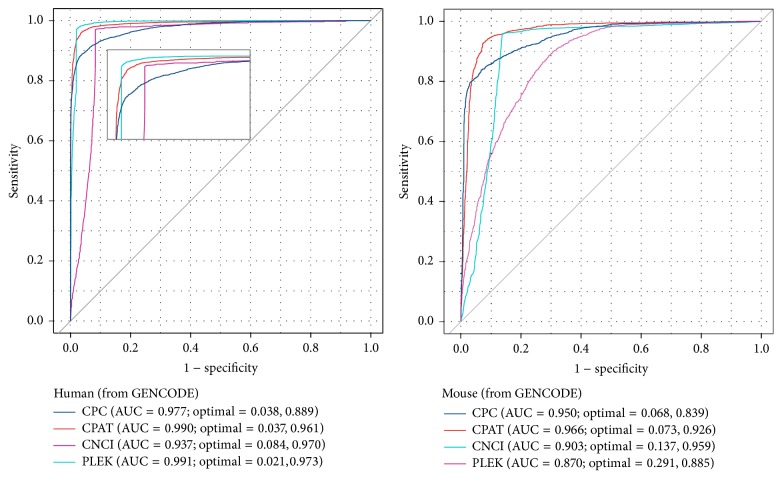
The ROC curves of CPC, CPAT, CNCI, and PLEK. We assessed the models using the same datasets as LncRNA-ID (selected from GENCODE) used. Both CPC and CPAT were evaluated with the latest versions.

ROC curves of CPC, CPAT, PLEK, and LncRNA-ID tested on human and mouse datasets were provided in [32]. Since CPC and CPAT are updated as the accumulation of gene database, it is useful to assess their performance with latest version and take CNCI into account. Here we utilise the test set of LncRNA-ID [32] (both the datasets of human and mouse are selected from GENCODE) to reevaluate CPC, CPAT, CNCI, and PLEK (Figure 2). According to [32], the area under curve (AUC) of LncRNA-ID on human dataset is 0.9829 (optimal = 0.0545, 0.9720), while on mouse it is 0.9505 (optimal = 0.0800, 0.9445) [32]. In our assessment, the performance of PLEK is identical with [32], while the performance of CPC and CPAT, as we anticipated, displayed some differences. The ROC curves were drawn in R with the package of pROC [71].

LncRScan-SVM is compared with CPC and CPAT on human and mouse datasets from UCSC (version hg19 of human and mm10 of mouse). CPC, as an excellent coding potential assessment tool, still achieves 98.37% of specificity on mouse testing dataset. CPAT, on the contrary, achieved the highest values of sensitivity both on the datasets of human and mouse. LncRScan-SVM surpasses CPAT with 89.20% and 89.14% of specificity on human and mouse datasets. For sensitivity, lncRScan-SVM obtained 93.88% and 95.29% on the same testing datasets, which are only 0.72% and around 0.1% lower than CPAT's results, respectively, but much higher than CPC's 67.23% and 75.46%. In addition, lncRScan-SVM also has the best results of accuracy and AUC [33] on these datasets.

For the same testing datasets, the running time of CPAT is the shortest and CPC shows the longest time to finish the process because of its alignment process. When being tested on a dataset containing 4,000 protein-coding and 4,000 long noncoding transcripts, CPAT takes 35.36 s and LncRNA-ID takes 65.35 s to accomplish the discrimination while PLEK and CPC need 21.47 m and 86.51 h, respectively [32]. PLEK is 8 times and 244 times faster than CNCI and CPC, respectively, on the same testing data [31], and lncRScan-SVM also needs about 10 times as much as CPAT to finish computation [33].

## 4. Application Scopes of the Methods

All these methods have own particular scopes to exert their talents, which means an appropriate program can help us obtain a satisfying result. The priority of utilising these tools under some particular circumstances is summarised in Table 4.

CPC is based on sequence alignment which facilitates protein-coding transcripts selection but impairs the performance of noncoding transcripts in that long noncoding transcripts share more similarities with coding transcripts such as putative ORF, which could mislead CPC. Also, because of alignment process, utilising CPC to analyse massive-scale data is a time-consuming process.

CPAT is also used to evaluate the coding potential, though the performance on long noncoding transcripts is acceptable. CPAT has a compromise between coding and noncoding transcripts that is not bad. Since the model of CPAT is logistic regression and the input file is FASTA format, CPAT is markedly superior in computational time which means CPAT is more suitable for being applied to data on a large scale. Furthermore, linguistic features make CPAT be able to analyse the sequences without annotation, and allowing users to train the model with their own dataset extends CPAT's scope of application. Users can apply CPAT to other species instead of being confined to human or mouse only.

CNCI is designed to distinguish between coding and long noncoding transcripts without the annotations of sequences. Because lots of lncRNAs are poorly annotated, this quality provided a more accurate discrimination for these sequences. CNCI is trained on human dataset but can also be applied to other mammals such as mouse and orangutan. CNCI displays acceptable results on vertebrates (except fish), but, for plants and invertebrates, the result is not very satisfying. CNCI is valuable when the sequences lack annotations or users do not have training set of other species. CNCI also shows a good performance when the transcripts are incomplete.

PLEK employs a higher fault tolerance algorithm and performs better when the sequences have indel errors. It is a proper tool to analyse the* de novo* assembled transcriptome datasets such as the sequences obtained from Roche (454) and Pacific Biosciences (PacBio) sequencing platforms. In addition to human and mouse, PLEK can also be used to other vertebrates and displays comparable results with the ones of CNCI. PLEK's model can be trained by users, but it takes a long time to be completed.

LncRNA-ID has many merits and delivers better all-round performance on human and mouse datasets. Although the time LncRNA-ID spent on classifying is nearly twice of CPAT, LncRNA-ID is still more efficient than other methods, which makes it a reasonable choice when data are on a massive-scale. The model of LncRNA-ID can be trained by users, but the most excellent attribute is the competence of handling the unbalanced training data. For studying those not well-explored species, LncRNA-ID takes priority when users have training datasets.

LncRScan-SVM achieves a good trade-off between the discrimination of coding and long noncoding genes. LncRScan-SVM is slower than CPAT and LncRNA-ID, but it is still acceptable. For analysing human and mouse datasets, lncRScan-SVM can be considered as a proper approach.

## 5. Discussion

According to the features selected by each tool, it is apparent that different tools have their own advantages and disadvantages. CPC is developed to assess coding potential of the transcripts; moreover, CPC is trained on datasets of protein-coding and noncoding RNA which means it achieves excellent performance when analysing ncRNAs. CPC provides a stand-alone version and a web server, but both of the two programs need vast amounts of time to process the sequences. As alignment-based tools, the performance of CPC varied when using different protein reference database. CPAT can present satisfying results efficiently partly because CPAT builds the logistic model which is faster than SVM. The web server of CPAT can display the result in an instant, which facilitates small scale prediction tasks. A minor disadvantage of CPAT is that the cutoff of CPAT varies from species to species and users have to determine the optimum cutoff value when they are training a new model. CNCI is designed to predict the transcripts assembled from whole-transcriptome sequencing data. Thus, CNCI offers a high accuracy on incomplete transcripts. CNCI did not provide result of elaborate comparison between CNCI and CPAT, but CPAT has no regard for the problem of incomplete transcripts. Meanwhile, UTRs of the transcripts may also interfere with the performance of CPAT. The features of ANT of CNCI closely resemble the hexamer of CPAT, but the distinguishing process of CNCI is more complicated and accurate than CPAT. However, the sliding window of CNCI slides 3 nt in each step, and consequently some deletion or frameshift errors may lead to a false shift and present users with a disappointing performance. In such cases, PLEK has made a considerable improvement and exhibits more flexibility when handling the indel sequencing errors. Indel errors are very common in the sequences obtained by today's sequencing platform, which means PLEK performs well for* de novo* assembled transcriptomes. With the indel error rate increasing, the accuracy of CNCI is decreasing while PLEK has no distinct fluctuation. Nonetheless, since the nucleotides compositions differ slightly among different species, the performance of PLEK on multiple species is not better than or approximately equivalent to CNCI whose performance is more stable on different species. Both LncRNA-ID and lncRScan-SVM achieve a balance between protein-coding and lncRNAs. But the capacity of lncRScan-SVM will be limited when analysing the sequences with a lack of annotation. Another point that needs to be brought up is that lncRScan-SVM and CNCI support*∗*.GTF as input file format.

It is apparent that nucleotides composition (such as *k*-mer and G + C content) and ORF are two classic and widely used feature groups. These features have strong discriminative power because protein-coding genes will finally be transcribed and translated to produce a specific amino acid chain, which requires some specified nucleotides composition and high-quality ORFs. As to the models of these tools, SVM (CPC, CNCI, and PLEK), logistic regression (CPAT), and random forest (LncRNA-ID) are more practical for lncRNA identification, though ANN or deep learning is a more popular machine learning algorithm now. Along with the protein-coding genes prediction, the annotations of lncRNA gene have been performed as well. A new tool named AnnoLnc (2015, available at http://annolnc.cbi.pku.edu.cn/index.jsp) has just been developed to annotate new discovered lncRNAs but related article has not yet been officially published. Users can access its web server for more information.

LncRNAs are receiving increasing attention and lncRNA identification has always been a challenge for researches of life science. For so many different types of sequences, various excellent tools should be developed to tackle different problems under various circumstances in the future. In this review, we summarised several tools for lncRNAs identification and concluded respective scopes. Due to their different scopes of application, using a method apposite to particular situation will be of essence to achieve convincing results. We hope this review can help researchers employ a more appropriate method in certain situations.

## Figures and Tables

**Table 1:** Overview of the methods concerning lncRNA identification.

	Published year	Testing datasets	Training species	Model	Query file format	Web interface
CPC	2007	ncRNA^*∗*^	Eukaryotic	SVM	FASTA	Yes
CPAT	2013	lncRNA^*∗*^	Human; mouse; fly; zebrafish	LR	BED; FASTA	Yes
CNCI	2013	lncRNA	Human; plant	SVM	FASTA; GTF	No
PLEK	2014	lncRNA	Human; maize	SVM	FASTA	No
lncRNA-MFDL	2015	lncRNA	Human	DL	*Unknown* ^*∗∗*^	*Unknown* ^*∗∗*^
LncRNA-ID	2015	lncRNA	Human; mouse	RF	BED; FSATA	No
lncRScan-SVM	2015	lncRNA	Human; mouse	SVM	GTF	No
LncRNApred	2016	lncRNA	Human	RF	FASTA	Web only

Testing datasets denote that one specific method is developed to discriminate ncRNAs or lncRNAs from protein-coding transcripts. The classification model of CPC, CNCI, PLEK, and lncRScan-SVM is support vector machine (SVM); CPAT employs logistic regression (LR); LncRNA-ID and LncRNApred utilise random forests (RF) and lncRNA-MFDL uses deep stacking networks (DSNs) of deep learning (DL) algorithm.

^*∗*^Note that the most popular tool CPC is trained and tested on datasets of ncRNAs and protein-coding transcripts. The training datasets of CPAT are also ncRNAs and protein-coding transcripts, though test on lncRNAs for CPAT is conducted and achieved a higher accuracy.

^*∗∗*^The access link of lncRNA-MFDL has expired; thus, we cannot verify the information that the original paper failed to mention.

**Table 2:** Summary of the features of each method selected.

	ORF	Codon	Sequence structure	Ribosome interaction	Alignment	Protein conservation
CPC	Quality; coverage; integrity	No	No	No	BLASTX	Number and *E*-value of hits; Distribution of hits
CPAT	Length; coverage	Hexamer Frequency	Content of the bases Position of the bases	No	No	No
CNCI	No	ANT matrix; Codon-bias	MLCDS	No	No	No
PLEK	No	No	Improved *k*-mer scheme	No	No	No
lncRNA-MFDL	Length; coverage	No	*k*-mer scheme Secondary structure MLCDS	No	No	No
LncRNA-ID	Length; coverage	No	Kozak motif	Ribosome release signal Changes of binding energy	Profile HMM based alignment	Score of HMMER Length of the profile Length of aligned region
lncRScan-SVM	No	Distribution of stop codon	Score of txCdsPredict; length of transcripts; length and count of exon	No	Phylo-HMM based alignment	Average PhastCons scores
LncRNApred	Length; coverage	No	Length of the sequence; signal to noise ratio; *k*-mer scheme; G + C content	No	No	No

All features are categorized into six groups according to the similarity or basic principles. Thus, some items in the table might not be exactly in one-to-one correspondence with the feature names given in the corresponding published references.

**Table 3:** Overview of each tool's performance on different testing datasets.

Testing dataset	CPC	CPAT	CNCI	PLEK	LncRNA-ID	lncRScan-SVM
*Human MCF-7 (PacBio)* ^*1*^						
Specificity	**99.90**		91.80	94.70		
Sensitivity	19.00		78.70	**95.80**		
Accuracy	**97.00**		91.30	94.70		
*Human HelaS3 (454)* ^*2*^						
Specificity	**99.90**		93.90	95.50		
Sensitivity	47.20		81.10	**92.50**		
Accuracy	**99.00**		93.70	95.40		
*Human (from GENCODE)* ^*3*^						
Specificity	**99.97**	99.55		89.18	95.28	
Sensitivity	66.48	86.95		**99.52**	96.28	
Accuracy	83.22	93.25		94.32	**95.78**	
*Mouse (from GENCODE)* ^*4*^						
Specificity	98.75	**98.95**		70.94	92.10	
Sensitivity	76.55	38.80		88.11	**94.45**	
Accuracy	87.65	68.88		79.49	**93.28**	
*Human (from GRCh37/hg19)* ^*5*^						
Specificity	**97.62**	85.28				89.20
Sensitivity	67.23	**94.60**				93.88
Accuracy	82.43	89.94				**91.94**
*Mouse (from GRCm38/mm10)* ^*5*^						
Specificity	**98.37**	88.17				89.14
Sensitivity	75.46	**95.34**				95.29
Accuracy	86.91	91.76				**92.21**

The results of the tools being tested on the same datasets are listed above. Bold numbers denote the highest value of the metrics.

^1^MCF-7 is available at http://www.pacb.com/blog/data-release-human-mcf-7-transcriptome/; ^2^dataset of HelaS3 is available at https://www. ncbi.nlm.nih.gov/sra/SRX214365; ^3,4^datasets are available at https://www.dropbox.com/sh/7yvmqknartttm6k/AAAQHvLZPjgjf4dtmHM7GNCqa/ H1_gencode?dl=0 and https://www.dropbox.com/sh/7yvmqknartttm6k/AACzaG-QJggvbXW6LA32oo7ba/M1_gencode?dl=0; ^5^dataset of human and mouse is available at http://journals.plos.org/plosone/article?id=10.1371/journal.pone.0139654.

**Table 4:** Priority of employing different methods on different situations.

	CPC	CPAT	CNCI	PLEK	LncRNA-ID	lncRScan-SVM
Coding potential assessment	✓	✓				
Human lncRNAs	✓	✓	✓	✓	✓	✓
Mouse lncRNAs		✓			✓	✓
Other Species^1^		✓	✓	✓	✓	
Testing data with sequencing errors^2^	✓		✓	✓		
Lack of annotation		✓	✓	✓		
Massive-scale data^3^		✓		✓	✓	✓
Trained by users^4^		✓		✓	✓	
Web interface	✓	✓				

This table only presents the preferences under different situations, which means a method with a tick can achieve a better performance under a certain circumstance.

^1^Only CPAT, LncRNA-ID, and lncRScan-SVM provide the model for mouse. When analysing other species, CPAT has the model for fly and zebrafish; CNCI and PLEK can predict the sequences of vertebrata and plant. CPAT, PLEK, and LncRNA-ID can build a new model based on users' datasets. ^2^Users can choose CNCI for incomplete sequences and CPC or PLEK for the transcripts with indel errors. ^3^CPAT is the most efficient method. Though lncRScan-SVM needs more time than CPAT and LncRNA-ID, it is also acceptable. ^4^LncRNA-ID can handle the imbalanced training data. Training PLEK with users' own datasets may be a time-consuming task.

## Additional Points

*Key Points*. (i) Different tools have different scopes. Users should select a proper tool according to the type of sequences. (ii) From the perspective of sequence types, CPC and CPAT are mainly used to assess coding potential. CNCI and PLEK can be applied to the sequences obtained from high-throughput sequencing platforms or the poorly annotated. LncRNA-ID and lncRScan-SVM are more accurate on human and mouse datasets. (iii) From the perspective of other functions, CPC and CPAT have web interfaces. The classification models of CPAT, LncRNA-ID, and PLEK can be trained on users' own datasets. CPAT, LncRNA-ID, and lncRScan-SVM can be utilised when the data to be analysed are on a massive-scale.
